# Utilization of Enclosure Space by Critically Endangered Musk Deer in Captivity

**DOI:** 10.3390/vetsci13030255

**Published:** 2026-03-09

**Authors:** Rongxin Li, Hong Ye, Xiaoping Lu, Qingxin Lv, Zisong Yang, Xiuxiang Meng

**Affiliations:** 1 School of Ecology & Environment, Renmin University of China, Beijing 100872, China; 2 School of Resource & Environment, Aba Teachers College, Wenchuan 623002, China

**Keywords:** Alpine musk deer (*Moschus chrysogaster*), captive conditions, shelters, influencing factors

## Abstract

Enclosure space constitutes the fundamental habitat for captive animals, directly influencing domestication success and production performance. Alpine musk deer (*Moschus chrysogaster*), an endangered ungulate present on the Tibet plateau and surrounding areas, has been commonly farmed in captivity as an essential strategy for the ex situ conservation and sustainable musk production. In captive musk deer farming, understanding spatial selection patterns within enclosures is critical for improving farming practices. To evaluate patterns of space use under captive conditions and the effect of enclosure designing, we studied 70 captive Alpine musk deer housed in 17 enclosures at the Zhuanglang Musk Deer Breeding Farm (Gansu, China) during the summer of 2022 (from July 1st to August 29th). Focal sampling and all-occurrence recording were adopted to collect the behavioral data of the musk deer, and the point occupancy rate was calculated to analyze the enclosure space utilization patterns. The results showed significant differences in activity site selection and shelter use time among captive musk deer (*p* < 0.05). The central enclosure site (G5), which contains the shelter, exhibited the highest utilization rate (21.21 ± 9.19%). Shelter use was significantly higher in adults (22.09 ± 7.80%) than in subadults (17.27 ± 3.98%) (*p* < 0.05), and significantly higher in males (29.55 ± 5.65%) than in females (20.86 ± 7.95%) (*p* < 0.05). However, at equal population density, the shelter use time between all-male and mixed-sex groups was not significant (*p* > 0.05). These results reveal that captive Alpine musk deer display distinct shelter use patterns, with shelters acting as key spatial resources. Therefore, this study provides practical implications for optimizing enclosure design (e.g., increasing shelter quantity and improving spatial distribution) to enhance the welfare and productivity of captive Alpine musk deer.

## Simple Summary

Musk deer are small forest animals native to Asia that produce musk, a naturally scented substance widely used in traditional medicine and perfumes. Due to excessive hunting and habitat loss, wild populations have declined sharply, and many musk deer are now kept on breeding farms for conservation and sustainable musk collection. However, living in fenced enclosures is very different from living in the wild, and limited space may affect the animals’ health and well-being. This study focused on the Alpine musk deer (*Moschus chrysogaster*), a mountain-dwelling species, to understand how individuals use space inside captive enclosures, especially shelters, which are covered structures that provide shade and protection. Researchers observed 70 musk deer at a breeding farm in China during summer, recording where the animals spent their time. The findings indicated that captive Alpine musk deer exhibited distinct spatial preferences across enclosure sites, with the highest utilization rate recorded for the central shelter area. Adult musk deer used shelters more than subadults, and male musk deer used shelters more than females, likely because males are more territorial with higher ranking in the social hierarchy. However, at equal population density, no significant difference was detected between all-male groups and mixed-sex groups. These findings demonstrate that shelters are essential resources for captive musk deer, serving as safe resting places that help them cope with heat and social stress. The findings suggest that breeding farms could provide more shelters in different areas of enclosures to reduce competition between animals, improve animal welfare, and ultimately support long-term conservation and sustainable musk production.

## 1. Introduction

Concealment behavior, a key animal survival strategy, reflects the selection and utilization of habitats by wild animals [1]. Concealed spaces are important resources for breeding and safe habitat, and wildlife usually prefers habitats with high concealment [2]. Under natural conditions, woodlands, shrubs, caves and densely vegetated vegetation constitute effective shelters, which can buffer environmental stressors (e.g., extreme weather) [3] and markedly lower predation risk [4]. In order to improve the survival rate of larvae, female individuals tend to select highly concealed habitats for rearing young [5].

Captive breeding has become a pivotal ex situ conservation strategy for endangered wildlife, providing opportunities for population recovery while ensuring sustainable resource utilization [6]. However, captive environments differ markedly from natural habitats. Enclosures are typically smaller, with heterogeneous resource distribution and higher animal density, which may intensify social stress and limit opportunities for solitude [7]. Under these conditions, animals often adjust their space-use strategies. For instance, captive sitatunga (*Tragelaphus spekii*) strongly preferred long-grass zones resembling wild habitats [8]; captive coyotes (*Canis latrans*) spent more time in sheltered areas to rest and remain vigilant [9]; and high-ranking Przewalski’s horses (*Equus ferus przewalskii*) monopolized key resource areas more than subordinates [10]. These examples illustrate that resource distribution within enclosures can shape spatial preferences and reinforce hierarchical competition.

Musk deer (*Moschus* spp.) are typical small, solitary, forest-type ruminants highly valued for the musk secreted by adult males, a substance traditionally used in Asian medicine and the perfume industry [11]. Overexploitation and habitat degradation have caused severe population declines, and musk deer are now listed in Appendix II of CITES and prioritized for protection in China [12,13]. Captive breeding is therefore a critical means to conserve these endangered species while ensuring sustainable musk utilization [14,15]. Among them, the Alpine musk deer is the most widely farmed species. In the wild, this species is highly solitary and territorial, with individuals maintaining largely exclusive home ranges [16,17]. In captivity, however, economic and spatial constraints necessitate group housing, confining individuals to enclosures far smaller than their natural home ranges and potentially altering their behavioral strategies [18]. Although limited evidence suggests that territoriality may persist under such conditions [19], systematic studies on spatial competition and site selection in captive musk deer remain lacking.

Based on these considerations, we hypothesized that Alpine musk deer exhibit distinct space-use patterns in captivity, with shelters acting as focal resources that influence enclosure use. Accordingly, this study aimed to (1) quantify patterns of spatial selection within enclosures and (2) evaluate the effects of age, sex, and group structure on shelter use. The findings provide insight into enclosure design and management practices that can enhance the welfare of captive musk deer.

## 2. Materials and Methods

### 2.1. Study Site and Period

The study was conducted from July to August 2022 at the Fengchun Alpine Musk Deer Breeding Farm in Zhuanglang County, Gansu Province, China. The farm is situated at the eastern extension of the Qilian Mountains and the western foothills of the Liupan Mountains, at an elevation of approximately 1800 m. The region has a temperate continental climate with four distinct seasons. The mean annual temperature is 8.1 °C, with the highest monthly average in July and the lowest in January. Annual precipitation averages 489 mm, most of which falls between July and September, and the frost-free period lasts about 142 days [17].

This study period was selected to coincide with the non-breeding season, thereby minimizing potential confounding effects associated with mating activities [20]. During this physiological stage, females are lactating while males undergo late-stage musk secretion and sachet maturation [21], constituting a critical phase for the captive herd.

### 2.2. Experimental Animals and Grouping

This study involved 70 captive Alpine musk deer (35 males and 35 females) distributed across 17 enclosures. These enclosures were selected based on two criteria: (1) observational accessibility, defined as unobstructed sightlines that allowed complete behavioral recording; and (2) herd composition, including group size, age structure, and sex ratio consistent with the experimental design. The experimental animals were divided into subadults (1 year old; *n* = 16 males) and adults (>1 year; *n* = 54, including 19 males and 35 females). Subadults were kept in all-male enclosures (four enclosures, four individuals each). Adults were housed either in all-male enclosures (two enclosures, four individuals each) or in mixed-sex groups (11 enclosures, nine with one male and three females, and two with one male and four females). All animals were fed twice daily within the indoor pens with a diet of commercial feed and wild-collected leaves. Enclosures were cleaned during feeding, and animals were otherwise left undisturbed.

### 2.3. Enclosure and Outdoor Yard

**Figure 1:**
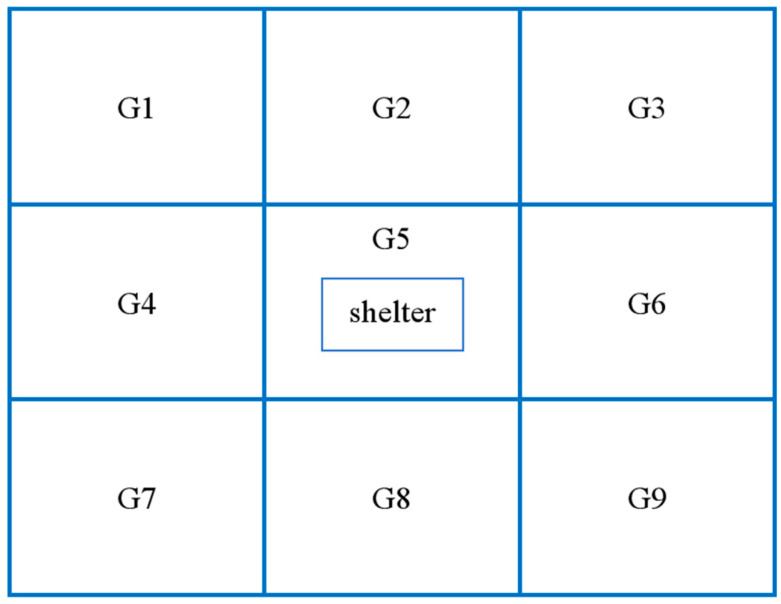
Spatial layout of the outdoor yard, illustrating functional zoning and central shelter placement for captive Alpine musk deer.

The farm covers approximately 10 ha and contains more than 250 enclosures. Each enclosure has a similar structure, consisting of 4–8 indoor pens and one outdoor yard (~200 m^2^). The outdoor yard is an open, continuous space with no physical barriers between sites. It is bounded by brick walls along the periphery, ensuring isolation from the external environment and adjacent enclosures. At the center of each yard, a brick shelter (2 m × 3 m × 1.5 m) was provided to serve as a refuge for resting and concealment, as well as to offer critical shade during high-temperature periods. The shelter had two entrances (front and back) but no through passage, and a solid partition divided the interior. The roof was made of impermeable concrete. For behavioral sampling, each outdoor yard was divided into a 3 × 3 grid (zones G1–G9), with the central cell (G5) containing the shelter (Figure 1).

### 2.4. Behavior Sampling

Behavioral observations were conducted during peak activity periods (06:00–10:00 and 16:00–20:00) [22]. Each animal was sampled twice per session (morning and evening), two days per week. Data were collected using focal animal sampling with continuous recording, documenting the time each individual spent in each grid cell and its movements between points. All individuals were identified by numbered plastic ear tags.

### 2.5. Data Processing

Behavioral samples were aggregated through the synthesis of behavioral sampling results. The point occupancy rate, defined as the ratio of the occupancy time at a specific point to the total sampling time, was computed for 17 enclosures. This metric was employed to characterize the utilization of the point by captive Alpine musk deer. The calculation formula is as follows:

$$Point\;occupancy\;rate=\frac{\;time\;spent\;at\;the\;point}{total\;sampling\;time}\times 100\%$$

### 2.6. Statistical Analysis

Friedman’s test and Wilcoxon signed-rank tests were used to evaluate differences in the utilization of grid points within the enclosure, determining the relative importance of the shelter for captive Alpine musk deer. The Kolmogorov–Smirnov test was used to test the normality of the data. If the data were normally distributed, T-tests were used to test the differences in the proportion of time occupied by Alpine musk deer at each point, from which the effects of age, sex, and community structure on the proportion of time spent in the shelter were judged. If the original data and the transformed data were non-normally distributed, the Mann–Whitney U test was applied to test the differences in the above data. Missing data were excluded from statistical analyses. The criterion for significance of differences was set at *p* = 0.05, and all data analyses were conducted with SPSS 26.0.

## 3. Results

### 3.1. Utilization Rate of Points in Captive Alpine Musk Deer Enclosure

The results showed that captive Alpine musk deer exhibited distinct spatial preferences in the enclosure, with the highest utilization rate (21.21 ± 9.19%) recorded for point G5. The utilization rate of each point in the enclosure is shown in Table 1.

Friedman’s test indicated significant variation among grid points (H = 22.632, *p* = 0.004, N = 55). Pairwise Wilcoxon comparisons showed that G5 differed significantly from G1, G3, G4, G6, and G9 (Table 2). These results confirm that shelters are the most frequently used resource within enclosures.

The analysis revealed that point G5 was significantly different from points G1, G3, G4, G6 and G9, and it had the highest value (21.21 ± 9.19%). Thus, point G5, which contains the shelter, represents a critical spatial resource for captive Alpine musk deer, and individuals spent a higher proportion of time using this area.

### 3.2. Shelter-Use Time and Age in Captive Alpine Musk Deer

**Figure 2:**
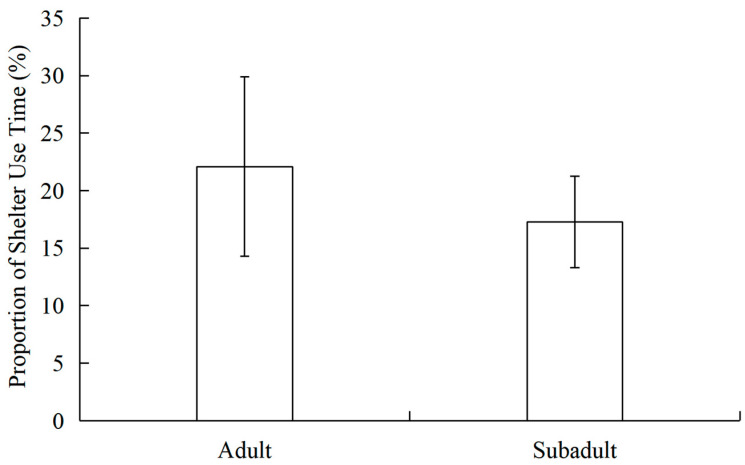
Differences in shelter use time between adult and subadult Alpine musk deer.

Shelter-use data were non-normally distributed (Kolmogorov–Smirnov test, *p* = 0.036). Mann–Whitney U test showed that adults spent significantly more time in shelters (22.09 ± 7.80%, N = 54) than subadults (17.27 ± 3.98%, N = 16; Z = −2.084, *p* = 0.037) (Figure 2).

### 3.3. Shelter-Use Time and Sex in Captive Alpine Musk Deer

**Figure 3:**
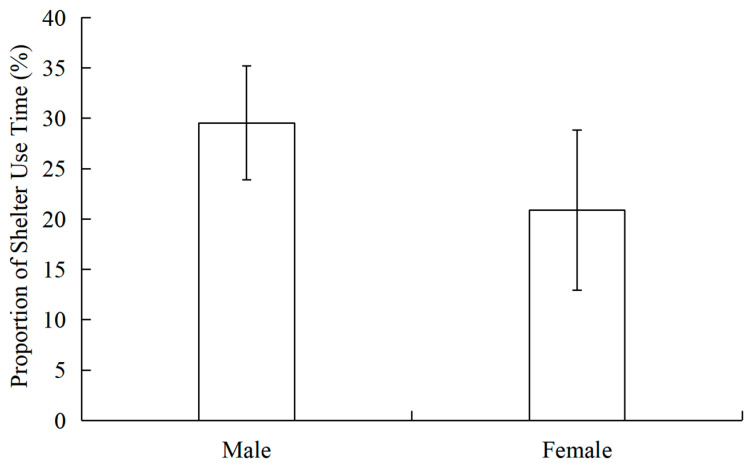
Differences in shelter use time between male and female Alpine musk deer.

The findings indicated that male musk deer had a significantly higher proportion of shelter use time (29.55 ± 5.65%, N = 9) than females (20.86 ± 7.95%, N = 27; t =3.020, *p* = 0.024) (Figure 3).

### 3.4. Shelter-Use Time and Community Structure in Captive Alpine Musk Deer

At equal population density, no significant difference was detected between all-male groups (20.54 ± 8.35%, N = 8) and mixed-sex groups (23.04 ± 8.29%, N = 36; Z = −0.761, *p* = 0.447).

## 4. Discussion

### 4.1. Selective Use of Enclosure Space

This study revealed that Alpine musk deer occupied the shelter-containing point (G5) significantly more than other sites, indicating its function as a prime territory within the enclosures. Concealed spaces are crucial for the survival of Alpine musk deer. In the field environment, Alpine musk deer show positive selectivity for habitat concealment [23], and their habitats are often located in areas with dense tree cover [24,25]. For ungulates, security is a key driver of habitat selection, and increased canopy cover effectively enhances their sense of safety [26]. In addition, Alpine musk deer are cold-adapted but heat-sensitive. Their dense, hollow pelage minimizes heat loss in winter but restricts heat dissipation in summer, and their active locomotion further increases metabolic heat production. As a result, high ambient temperatures and limited water availability become the primary summer stressors, making thermoregulation and hydration key determinants of habitat selection [23].

Habitats with high concealment offer abundant water sources and sheltered environments for animals. The comparative analysis demonstrated that Alpine musk deer still preferred shelters with high shade cover in captivity. Unlike natural habitats, captive enclosures offer limited movement space, which forces increased inter-individual interactions among musk deer. In contrast to other grids, the shelter in G5 provides a concealed space for the musk deer to avoid conspecifics and alleviate social stress. Moreover, the sampling time of this experiment was in summer. Occupying the shelter allows Alpine musk deer to avoid external heat and access cooler resting areas, making G5 the most utilized grid and highlighting its ecological importance.

Meanwhile, the strongly solitary and territorial nature of Alpine musk deer leads to their exclusive use of shelters in captivity. Within their natural habitat, Alpine musk deer typically have a home range of 23.78 hm^2^ in summer [16] and up to 15–32 hm^2^ in winter [27], which they defend exclusively. Although the farming of musk deer in China began in the 1950s, captive musk deer have still not been fully domesticated despite more than 60 years [28]. Even in restricted group housing, solitary behavior is largely retained [19]. Territoriality in captivity reflects individual behavioral adaptations to environmental variation [29]. Higher territoriality is associated with increased vigilance and shyer behavioral traits, as indicated by a tendency to seek dry, sheltered, and secure microhabitats for resting and ruminating [25]. Within enclosures, shelters serve as critical resource hubs, providing access to water and solitude, rendering them the most intensively utilized microsites.

Within the enclosure, G5 held the highest importance value, yet its utilization rate did not differ significantly from those of G2, G7, or G8. This may be due to the fact that G7 is located against the wall, providing a shelter space similar to the shelter. Furthermore, the angle of sunlight falling on the yard allowed G7 to provide a broader shaded area. G2 and G8 are located in the middle of the outdoor yard, which is an important gateway to other points, leading musk deer to spent a relatively high proportion of their time occupying these three points.

### 4.2. Influencing Factors of Shelter Use in Captive Musk Deer

Statistical analysis revealed significant age-related differences in the use of sheltered spaces by captive Alpine musk deer, with adults spending a significantly greater proportion of time in sheltered spaces compared to subadults. Studies have shown that hierarchical ordering among adults is more stable than among subadults [30]. Moreover, social hierarchies among animals strengthen with prolonged cohabitation, and clear dominance structures are more likely to emerge in groups that remain stable over time [31]. Adults and subadult musk deer were kept in separate outdoor enclosures. After weaning, subadults are separated from maternal females and relocated to new enclosures. Within these newly formed cohorts, clear dominance hierarchies remain unestablished, and resource-holding potential is less stable compared with adult Alpine musk deer. Consequently, subadults exhibit a significantly lower proportion of time allocated to shelter occupation. In addition, immature subadults are more physically active and curious [32], often manifesting in frequent play and exploration [33]. Therefore, subadult Alpine musk deer exhibit more extensive movement in the enclosure and do not concentrate on the G5 point position. In contrast, adults spend more time and energy on activities such as maintaining territories and reproduction [34]. This behavioral pattern may have resulted in adult Alpine musk deer allocating extended durations to resting within shelters. Among other animals, captive giant panda (*Ailuropoda melanoleuca*) subadults showed significantly more active behavior than adults [35], and captive American bison (*Bison bison*) subadults explored their enclosures more extensively [36], consistent with our findings. Compared to adults, subadults are less experienced in survival and have lower risk perception [37], reducing their use of shelters.

In most mammals, males monopolize more resources than females, and our findings suggest that this male-biased resource control persists under captive conditions. Male musk deer displayed greater resource-holding potential than females and consequently exhibited stronger territoriality. Previous research also indicates that, for a given resource, males tend to exploit it more intensively than females [14]. In this study, shelters can be considered as an important resource in the enclosure, consistent with the findings of related studies. The larger body size and higher aggressiveness of males [38], together with higher dominance rank [39], reduce the likelihood that females succeed in resource competition. To minimize the risk of conflict and injury, subordinate females often adopt avoidance strategies, voluntarily relinquishing resources to dominant males [38]. This explains why male musk deer in this study spent a greater proportion of time occupying shelters. In the wild, male control of resources sustains and attracts females for mating, encouraging males to invest more effort in occupying and maintaining these resources [40]. This study was conducted during the musk-secreting period (July–August), when male musk deer show reduced food intake and overall activity [29]. This behavioral shift increases resting behaviors to minimize energy expenditure and facilitate energy storage in preparation for musk production and the subsequent breeding season [32], likely contributing to males spending more time in shelters.

In this study, the proportion of time spent in G5 shelters did not differ significantly between all-male and mixed-sex groups. This may reflect behavioral flexibility in captivity, where subordinate individuals suppress territorial behavior and avoid direct conflict to mitigate the risk of injury and unnecessary energy expenditure during resource competition [19]. Thus, the community structure has no significant effect on the proportion of time that captive Alpine musk deer spend occupying shelters.

## 5. Conclusions and Recommendations

This study demonstrates distinct patterns of spatial utilization in captive Alpine musk deer, with centrally located shelters functioning as critical resources that provide thermoregulatory benefits and mitigate social stress. To reduce competition and enhance welfare, enclosures should include multiple, evenly distributed shelters exceeding group size. Additionally, mixed housing of adults and subadults could be considered to alleviate crowding and promote more balanced space use.

## Figures and Tables

**Table 1:** Proportion of time occupied by captive Alpine musk deer on each point of the outdoor yard.

Point	Proportion (%)	Point	Proportion (%)
G1	14.31 ± 9.84	G6	15.40 ± 8.45
G2	19.02 ± 12.83	G7	17.04 ± 10.20
G3	16.80 ± 8.35	G8	19.34 ± 12.10
G4	16.29 ± 8.48	G9	14.42 ± 7.55
G5	21.21 ± 9.19		

**Table 2:** *p* for pairwise comparisons of time proportion occupied by each site.

	G1	G2	G3	G4	G5	G6	G7	G8	G9
G1		0.006 *	0.003 *	0.012 *	0.000 *	0.009 *	0.011 *	0.015 *	0.074
G2			0.214	0.910	0.063	0.744	0.722	0.522	0.213
G3				0.919	0.025 *	0.466	0.350	0.732	0.248
G4					0.021 *	0.300	0.527	0.861	0.580
G5						0.002 *	0.091	0.121	0.001 *
G6							0.538	0.325	0.561
G7								0.913	0.376
G8									0.170

Note: *, significant difference *p* < 0.05 .

## Data Availability

The original contributions presented in this study are included in this article. Further inquiries can be directed to the corresponding author(s).
